# Examining Causal Pathways to Suicidal Ideation and Nonsuicidal Self‐Injury in the Adolescent Brain Cognitive Development Study

**DOI:** 10.1111/sltb.70068

**Published:** 2025-11-24

**Authors:** Marvin Yan, Erich Kummerfeld, Eric Rawls, Kathryn R. Cullen, Bonnie Klimes‐Dougan

**Affiliations:** ^1^ Department of Psychology University of Minnesota Minneapolis Minnesota USA; ^2^ Institute for Health Informatics University of Minnesota Minneapolis Minnesota USA; ^3^ Department of Psychology University of North Carolina Wilmington Wilmington North Carolina USA; ^4^ Department of Psychiatry and Behavioral Sciences University of Minnesota Minneapolis Minnesota USA

**Keywords:** adolescents, causal discovery analysis, neurocognition, neuroimaging, nonsuicidal self‐injury, suicide

## Abstract

**Introduction:**

Suicide is the second leading cause of death in adolescents in the United States. There is an urgent need to advance understanding of risk mechanisms in adolescents to guide early interventions. While prior research has implicated cognition, neural connectivity, and psychopathology in relation to adolescent suicidal ideation (SI) and nonsuicidal self‐injury (NSSI), there is a relative lack of clarity regarding the causal structure of these factors, particularly in early adolescence.

**Methods:**

Causal discovery analysis was applied to neuroimaging, neurocognition, and clinical assessment data from the baseline visit of the Adolescent Brain Cognitive Development Study when the participants were 9–10 years old (*N =* 8937; 49.6% female) to produce models of causal relationships.

**Results:**

In the discovered model, causal pathways from resting state functional connectivity to externalizing and internalizing psychopathology were observed. Greater externalizing psychopathology increased SI and NSSI. Cognitive performance indirectly increased SI and NSSI via its negative relationship with externalizing psychopathology. Finally, more SI increased NSSI.

**Conclusions:**

In this developmental window prior to when the risk of suicide accelerates, it is critical to begin to advance our understanding of the processes that may undergird suicide risk (neural, cognitive performance), features of psychopathology and the potential progression of SI and NSSI (both risk factors for suicide). Future research should incorporate other factors related to SI and NSSI to produce a more comprehensive understanding of the mechanisms of risk. This line of research has the potential for a more comprehensive understanding of risk and provides avenues for prevention.

## 1 Introduction

Suicide is the second leading cause of death among adolescents aged 10–14 years old (CDC 2022). Research on adolescent suicide has continued to rise, yet the ability to predict suicide has not meaningfully improved for quite some time (Franklin et al. 2017). While the period of adolescence is often defined as between ages 10 and 19 (WHO, n.d.), studies thus far have largely focused on middle to late adolescence. Because the research on adolescent SITBs has focused on mid‐ to late‐adolescence, less is known about SITBs during early adolescence. There is evidence that SITBs are more common during mid‐adolescence (Muehlenkamp et al. 2018), which may be due in part to concurrent social and neurodevelopmental changes (Casey et al. 2010) that start during early adolescence and become more pronounced throughout adolescence. It is possible that early adolescence represents a unique developmental context that has different pathways to SITBs when compared to older adolescents. Thus, efforts to examine possible causal mechanisms of suicidal risk early in adolescence, before suicide risk rises, are a priority. Additionally, although suicide is a complex issue, much of the existing research has examined a singular domain of risk. A multimodal approach that leverages machine learning to understand suicide risk may untangle the complexity of causal pathways to suicidal behaviors, particularly in young adolescents.

Adolescence is a key developmental period where the onset of psychiatric illnesses is most common (Solmi et al. 2022). Both NSSI and SI can manifest during adolescence as maladaptive behavioral and cognitive coping mechanisms. Each year, approximately 13.4% of adolescents engage in at least one instance of SI (SAMHSA 2024). While SI does not always progress to a suicide attempt (SA), SI is nonetheless associated with SA (McHugh et al. 2019). Another important risk factor for suicide is nonsuicidal self‐injury (NSSI). It is estimated that 19% of adolescents will engage in NSSI in a given year (Lim et al. 2019). Although NSSI has been conceptualized as a separate process from SI and suicidal behaviors due to a lack of suicidal intent, research suggests that NSSI is strongly associated with both SI and SA (Nock et al. 2006). Left unaddressed, SI and NSSI are linked to worse broad mental health outcomes in adulthood (Copeland et al. 2017; Daukantaitė et al. 2021), which can also be risk factors for SA.

### 1.1 Theoretical Models of NSSI and SI

While existing research has established a relationship between NSSI, SI, and SA, the causal nature of these relationships is unclear. Whether NSSI confers risk for later SI, or is a consequence of pre‐existing SI, requires further examination. The field is moving towards an ideation‐to‐action framework that requires additional processes to occur before SI can progress to SA (Klonsky and May 2014). Here, NSSI can be viewed as one way of acquiring the capability for suicide due to its association with higher pain tolerance, lowered fear of death, and its tendency to be mechanically similar actions that could be used in an SA (Van Orden et al. 2010; Willoughby et al. 2015). Accordingly, suicidal thoughts may lead to actions, possibly including engagement in NSSI, which could then in turn result in higher risk of a suicide attempt and death by suicide. Another framework, the emotional cascade model, suggests that intense, negative rumination can lead to a level of emotional dysregulation whereby individuals seek maladaptive behavioral coping mechanisms such as NSSI to relieve their distress (Selby and Joiner 2013). The ideation‐to‐action and emotional cascade models are similar in that they theorize that SI is causal of, or at least present prior to NSSI.

It is important to acknowledge the possibility that SI and NSSI are associated but no direct causal relationship exists between them (i.e., some confounding variable may be causing both NSSI and SI). It is also possible that NSSI can cause SI. Of note, relatively few studies have tested whether NSSI is predictive of SI, highlighting the need to also test this relationship (Ribeiro et al. 2016). There is some evidence that aspects related to NSSI, such as physical scarring, can predict SI in young adults (Burke et al. 2016). Other research with adolescents has shown that NSSI itself can predict later SI (Guan et al. 2012). While the discussion of the relationship between SI and NSSI thus far has occurred in the context of ultimately determining risk for SA, the current study did not include SA in the model due to infrequent endorsement, which is to be expected for young adolescents. Thus, all further discussion of self‐injurious thoughts and behaviors (SITBs) in the current study will refer to only NSSI and SI.

### 1.2 SITB‐Related Factors

It is also important to consider the effects of relevant factors on SITBs because of their tendency to modulate risk for SITBs in adolescents. While internalizing psychopathology has been more frequently linked to SITBs (Kranzler et al. 2016; Verona and Javdani 2011), there is growing evidence that externalizing psychopathology is also implicated in SITBs (Clapham and Brausch 2024; Meszaros et al. 2017), particularly in this age group. While SITBs are composed of behavioral components, they are also deeply rooted in cognitive resources. More specifically, prior research has identified differences in episodic memory, attentional bias towards self‐injury stimuli, specific aspects of impulsivity such as negative urgency, along with general cognitive performance in adolescents with SITBs (Goreis et al. 2024; Hamza et al. 2015; Huber et al. 2022; Mürner‐Lavanchy et al. 2022). It is important, however, to note that this evidence supporting the link between cognitive performance and SITBs is mixed, particularly in adolescents (Cha et al. 2019). These cognitive processes have neural underpinnings. Indeed, SITB‐related adolescent resting state functional magnetic resonance imaging (rsfMRI) studies have found aberrations in brain regions implicated in self‐referential thought and rumination, such as the default mode network (DMN; Zhou et al. 2020), and orientation of attention towards important stimuli (e.g., threat), such as the salience network (Schimmelpfennig et al. 2023). Additionally, differences in regulatory regions of the brain such as the frontoparietal, and central executive networks have been observed in adolescents engaging in SITBs (Auerbach et al. 2021; Cao et al. 2021; Wiglesworth et al. 2023). Taken together, this suggests that SITBs are associated with neurobiology, cognition, and psychopathology. More research is needed to determine the causal nature of the relationships between these factors.

### 1.3 Sex Differences in SITBs and SITB‐Related Factors

There are documented sex differences in SITBs and factors related to SITBs. First, there is evidence that NSSI is more prevalent in males vs. females during late childhood and early adolescence; however, this pattern is flipped during mid‐adolescence such that females are more likely than males to engage in NSSI (Moloney et al. 2024). Similarly, more males vs. females reported engaging in SI during early adolescence (Huber et al. 2022). Additionally, there is a trend such that females are more likely to report symptoms of internalizing psychopathology while males are more likely to report symptoms of externalizing psychopathology (Chaplin and Aldao 2013). Taken together, prior research suggests that there are important differences during early adolescence, especially in psychopathology and SITBs, which may result in unique pathways of risk for males vs. females.

### 1.4 Causal Discovery Analysis

Causal discovery analysis (CDA) is a set of data‐driven analytic techniques that use machine learning algorithms to produce models containing causal relationships between pairs of variables. Briefly, CDA takes in a set of variables and constructs a graph of paths between variables, orienting the directions of as many of the paths as possible based on a set of causal assumptions (Glymour et al. 2019). Thus, the output of CDA is a model that contains statistically plausible causal relationships between variables. A variety of CDA algorithms exist that have good performance in recovering the structure of the graph and orienting the directions of the paths. CDA is particularly well‐suited for psychopathology research because it can be applied to observational and cross‐sectional data. Thus, CDA‐identified causal relationships can elucidate potential targets for intervention and possible underlying mechanisms of psychological disorders, including the contributions of proximal associated factors. Previous studies have applied CDA to various areas of psychopathology (Camchong et al. 2023; Miley et al. 2023; Rawls et al. 2021); however, no published research has used CDA to better understand SITBs.

### 1.5 Study Goals

The goal of the current study was to produce causal models of SI and NSSI by leveraging CDA in a large, nationally representative dataset of early adolescents. To identify both proximal and distal causal factors for SI and NSSI, CDA was used to explore potential causal influences including neural connectivity, cognitive performance, and broad symptoms of psychopathology. It was hypothesized that a top‐down structure would be observed such that neural connectivity would causally impact cognitive performance, which would have downstream causal effects on psychopathology, ultimately increasing the risk of SI and NSSI in this sample. In addition to the causal effects these relevant factors may have on SITBs, SI and NSSI may also have causal effects on one another. Thus, we were particularly interested in any causal pathways between NSSI and SI that may be identified by the models. Although several theoretical models suggest that SI causes NSSI, it is possible that fewer studies have tested the relationship in the opposite direction or there is an underlying variable confounding this relationship. Given these possibilities, we did not have an a priori hypothesis regarding the direction of the relationship between SI and NSSI. Due to the evidence of consistent sex differences in many of the variables, we conducted sensitivity analyses to examine the moderating effect of assigned sex at birth by running CDA on male and female participants separately. We hypothesized that internalizing psychopathology would be causally linked to SITBs in the model of females while externalizing psychopathology would be causally linked to SITBs in the model of males. Clarifying the causal roles that risk factors (i.e., neural connectivity, cognitive performance, and psychopathology) and SITBs themselves (i.e., SI and NSSI) play in risk pathways for SITBs will hopefully elucidate targets for intervention. It is important to conduct this work in early adolescents, before the risk for SITBs and suicide rises dramatically during mid to late adolescence (Moloney et al. 2024), because results can inform SITB risk screening processes and prevention efforts in addition to interventions for SITBs that are already present.

## 2 Materials and Methods

### 2.1 Data

#### 2.1.1 Sample

The sample for the current study was drawn from the 5.1 Data Release of the Adolescent Brain Cognitive Development (ABCD) Study (Barch et al. 2017). The ABCD Study is a large, longitudinal study that conducts annual multimodal assessments on adolescents starting between 9 and 10 years old. Informed consent and assent were obtained from parents and adolescents at their respective site. Each site acquired institutional review board approval either locally or centrally from the University of California, San Diego's institutional review board (Auchter et al. 2018). The current study focused on the baseline assessment of the ABCD Study, which consisted of 11,868 research participants. After performing listwise deletion in preparation for CDA and excluding research participants whose neuroimaging data did not meet the ABCD Study's recommended quality control standards (see Supporting Information), the final sample included 8937 participants (49.6% female at birth, *N* = 4434) that were 9 and 10 years old (*M* = 9.92, SD = 0.62). A full description of the sample characteristics is provided in Table 1.

**TABLE 1:** Sample characteristics.

	Full sample (*N* = 8937)	Male (*N* = 4503)	Female (*N* = 4434)
	*N* (% of full sample)		
**Race**			
White	6780 (75.9)	3470 (38.8)	3310 (37.0)
Black	1770 (19.8)	848 (9.5)	922 (10.3)
Asian	557 (6.2)	256 (2.9)	301 (3.4)
American Indian or Alaskan Native	304 (3.4)	149 (1.7)	155 (1.7)
Native Hawaiian or Pacific Islander	54 (0.6)	30 (0.3)	24 (0.3)
**Combined family income**			
Less than $5000	256 (2.9)	133 (1.5)	123 (1.4)
$5000–$11,999	277 (3.1)	136 (1.5)	141 (1.6)
$12,000–$15,999	205 (2.3)	103 (1.2)	102 (1.1)
$16,000–$24,999	363 (4.1)	192 (2.1)	171 (1.9)
$25,000–$34,999	495 (5.5)	229 (2.6)	266 (3.0)
$35,000–$49,999	698 (7.8)	348 (3.9)	350 (3.9)
$50,000–$74,999	1133 (12.7)	580 (6.5)	553 (6.2)
$75,000–$99,999	1223 (13.7)	600 (6.7)	623 (7.0)
$100,000–$199,999	2573 (28.8)	1327 (14.8)	1246 (13.9)
$200,000 and greater	993 (11.1)	492 (5.5)	501 (5.6)
**Self‐injurious thoughts and behaviors**			
Nonsuicidal self‐injury	343 (3.8)	206 (2.3)	137 (1.5)
Suicidal ideation	273 (3.1)	156 (1.7)	117 (1.3)
	**Mean (SD)**		
**Age in years**	9.94 (0.63)	9.95 (0.63)	9.92 (0.62)
**Child Behavior Checklist** ^a^			
Internalizing	48.3 (10.6)	49.1 (10.5)	47.4 (10.5)
Externalizing	45.4 (10.2)	46.1 (10.6)	44.7 (9.8)
**Cognitive performance** ^a^	101.5 (17.6)	101.3 (17.6)	101.6 (17.6)

*Note:* Demographic and clinical characteristics of the final sample. Participants could endorse multiple racial categories. Combined family income information was missing for 721 (8.1%) participants.

^a^ Cognitive performance and Child Behavior Checklist scores were already standardized based on participant age (see Supporting Information).

### 2.2 Measures

Neuroimaging measures in the current study included eyes‐open rsFMRI data. The ABCD Study provides tabulated data using predefined networks according to the Gordon network atlas (Gordon et al. 2016). The current study focused on the resting state functional connectivity (rsFC) between several networks of interest that were identified based on prior studies that examined rsFMRI indices of SITBs in adolescents (Auerbach et al. 2021; Cao et al. 2021; Lewis et al. 2025). These included the default, frontoparietal, cingulo‐opercular, cingulo‐parietal, and salience networks as defined by the Gordon network atlas.

As a measure of cognitive performance, the composite score from the NIH Toolbox (Gershon et al. 2013) was used. The NIH Toolbox included seven different cognitive tasks (Picture Vocabulary, Flanker Inhibitory Control and Attention, Picture Sequence Memory, Dimensional Change Card Sort, Pattern Comparison Processing Speed, Oral Reading Recognition, List Sorting Working Memory). This score represents a participant's performance across various domains of neurocognition, including language and reading skills, executive functioning, episodic memory, processing speed, and working memory (Gershon et al. 2013) and is standardized based on the participant's age (see Supporting Information).

To obtain an empirically derived measure of psychopathology, the Child Behavior Checklist (CBCL) was included (Achenbach 1999). The CBCL is a 113‐item parent‐reported measure of psychological symptoms observed in the adolescent. The internalizing and externalizing psychopathology subscale standard scores (i.e., T‐scores) were used in the models.

NSSI and SI were based on the updated version of the Kiddie Schedule for Affective Disorders and Schizophrenia for DSM‐5 (Kaufman and Schweder 2004). If current engagement was endorsed, then NSSI and SI were coded as present, and absent otherwise. Due to concerns of underreporting and inconsistent reporting (Klimes‐Dougan 1998), the history of these thoughts and behaviors was coded as present if either the adolescent or the parent endorsed current engagement.

### 2.3 Data Analytic Plan

#### 2.3.1 CDA

To perform the CDA, the Tetrad graphical user interface 7.5.0 was used (Ramsey et al. 2018). The CDA algorithm selected in the current study was Greedy Fast Causal Inference (GFCI) due to its performance on large datasets and its ability to represent and discover unmeasured common causes (Ogarrio et al. 2016).

Briefly, GFCI starts with an empty graph and discovers edges between variables that would best improve the model fit score being used. Then, GFCI orients the direction of the edges using conditional independence rules (see Supporting Information). Structural equation modeling was then used to estimate standardized edge weights. The following settings for GFCI in Tetrad were used: the penalty discount for the Bayesian Information Criterion (i.e., the model fit score) was set at 1, the cutoff value of edges was set at *p* = 0.01, and the maximum degree of the graph was set at 1000.

No background knowledge structure was supplied to the GFCI algorithm and it was allowed to freely discover connections between any of the variables. Although the CBCL asks parents to report on their child's symptoms over the past 6 months, the internalizing and externalizing subscales are highly stable over a period of 12 months (*r* = 0.80 for internalizing and *r* = 0.82 for externalizing; Achenbach and Rescorla 2001). Therefore, we decided to treat all of the variables as concurrent measures of functioning at the baseline visit of the ABCD Study and did not restrict the types of connections the algorithm could discover.

## 3 Results

The results here focus on causal pathways leading to SI and NSSI. Findings from the complete models, along with confidence intervals for all of the pathways, will be presented in Tables S1–S3. Finally, analyses using lifetime SITBs are also presented in Figures S1–S3.

### 3.1 Initial Findings

**FIGURE 1:**
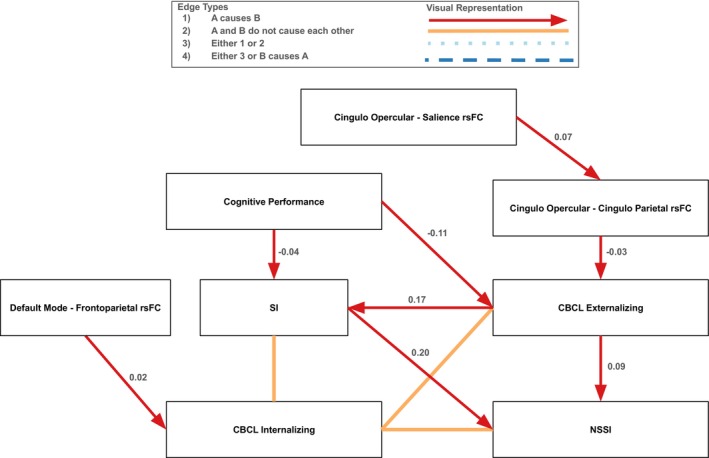
Causal pathways to SI and NSSI in the full sample. Portion of the graph displaying causal pathways to SI and NSSI in the full sample. For the full graph for the full sample, see Figure S1. Red arrows indicate a directional causal relationship was identified, orange solid lines indicate that the two variables did not cause each other because at least one latent confounding variable was discovered, light blue dotted lines indicate that either a causal relationship exists in one direction or a latent confounding variable was discovered, and dark blue dashed lines indicate that the relationship between the two variables is unclear (i.e., there could be a directional causal relationship in either direction or there could be a latent confounding variable). Standardized edge weights calculated using structural equation modeling are displayed next to each edge. All edges were significant at the *p* = 0.01 level.

In the full sample, the GFCI model discovered causal pathways from neural connectivity indices and psychopathology to SITBs (Figure 1). One path was cingulo‐opercular and salience network rsFC → cingulo‐opercular and cingulo‐parietal network rsFC → externalizing psychopathology. Here, greater cingulo‐opercular and salience network rsFC increased cingulo‐opercular and cingulo‐parietal network rsFC (*β* = 0.07), then greater cingulo‐opercular and cingulo‐parietal network rsFC decreased externalizing psychopathology (*β* = −0.03). More externalizing psychopathology then separately increased SI (*β* = 0.17) and NSSI (*β* = 0.09). Of note, greater SI increased NSSI (*β* = 0.20); therefore, all pathways that lead to SI also lead to NSSI. Thus, externalizing psychopathology was a proximal risk factor for SITBs, while cingulo‐opercular and salience network rsFC, along with cingulo‐opercular and cingulo‐parietal network rsFC, were protective against SITBs by decreasing externalizing psychopathology indirectly and directly. Additionally, higher cognitive performance decreased externalizing psychopathology (*β* = −0.11) and SI (*β* = −0.04). Therefore, higher cognitive performance was also directly and indirectly protective of SITBs by decreasing externalizing psychopathology and SI. Moreover, latent confounding variables were discovered such that internalizing psychopathology did not have causal pathways to externalizing psychopathology, SI, or NSSI, and did not have any causal pathways coming from externalizing psychopathology, SI, or NSSI.

### 3.2 Follow‐Up Analyses

We conducted separate models for males and females, given the notable sex differences in patterns of psychopathology and SITBIs. Although there is emerging evidence linking externalizing psychopathology to SITBs, there is considerable evidence supporting the relationship between internalizing psychopathology and SITBs (Dervic et al. 2008). Importantly, there were approximately 48.9% and 35.1% more males than females that reported NSSI and SI, respectively. Given the overrepresentation of males in those that endorsed SITBs and externalizing psychopathology, which is typically more commonly observed in males (Matos et al. 2017), it was possible that sex assigned at birth was affecting the initial results.

#### 3.2.1 Males

**FIGURE 2:**
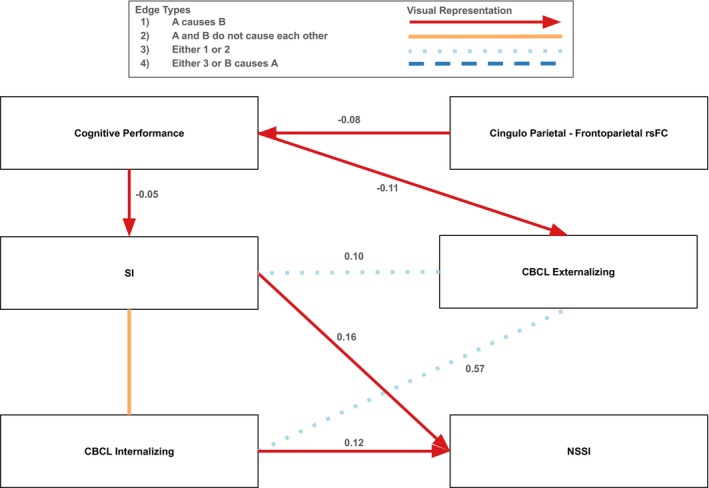
Causal pathways to SI and NSSI in the male subsample. Portion of the graph displaying causal pathways to SI and NSSI in the male subsample. For the full graph for the male sample, see Figure S2. Red arrows indicate a directional causal relationship was identified, orange solid lines indicate that the two variables did not cause each other because at least one latent confounding variable was discovered, light blue dotted lines indicate that either a causal relationship exists in one direction or a latent confounding variable was discovered, and dark blue dashed lines indicate that the relationship between the two variables is unclear (i.e., there could be a directional causal relationship in either direction or there could be a latent confounding variable). Standardized edge weights calculated using structural equation modeling are displayed next to each edge. All edges were significant at the *p* = 0.01 level.

In males, there was a causal pathway such that cingulo‐parietal and frontoparietal network rsFC → cognitive performance → SI → NSSI (Figure 2). More specifically, greater cingulo‐parietal and frontoparietal network rsFC decreased cognitive performance (*β* = −0.08), higher cognitive performance decreased SI (*β* = −0.05), and more SI increased NSSI (*β* = 0.16). Similar to the full sample, greater cognitive performance was protective against SITBs by decreasing SI, which had the indirect effect of also decreasing NSSI. Also, greater cingulo‐parietal and frontoparietal network rsFC conferred risk for SITBs through decreasing cognitive performance. An additional pathway was observed such that more internalizing psychopathology increased NSSI (*β* = 0.12). Of note, there were a couple of pathways that were not definitively oriented. It is possible that the connection from internalizing psychopathology to externalizing psychopathology is a unidirectional path from internalizing to externalizing psychopathology (*β* = 0.57), or there is an unmeasured latent confounder such that there are no causal pathways between internalizing and externalizing psychopathology. Similarly, the connection between externalizing psychopathology and SI could be a unidirectional path from externalizing psychopathology to SI (*β* = 0.10), or there is a latent confounder such that there are no causal pathways between these two variables. Finally, there were unmeasured confounding variables such that there were no causal pathways between internalizing psychopathology and SI.

#### 3.2.2 Females

**FIGURE 3:**
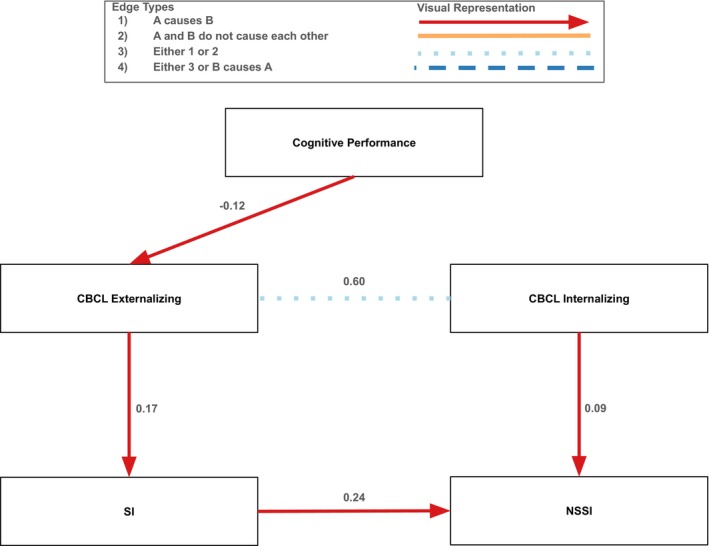
Causal pathways to SI and NSSI in the female subsample. Portion of the graph displaying causal Pathways to SI and NSSI in the female sample. For the full graph for the female sample, see Figure S3. Red arrows indicate a directional causal relationship was identified, orange solid lines indicate that the two variables did not cause each other because at least one latent confounding variable was discovered, light blue dotted lines indicate that either a causal relationship exists in one direction or a latent confounding variable was discovered, and dark blue dashed lines indicate that the relationship between the two variables is unclear (i.e., there could be a directional causal relationship in either direction or there could be a latent confounding variable). Standardized edge weights calculated using structural equation modeling are displayed next to each edge. All edges were significant at the *p* = 0.01 level.

In females, there was one causal pathway between neurocognition and SITBs (Figure 3). The pathway was: cognitive performance → externalizing psychopathology → SI → NSSI. More specifically, higher cognitive performance decreased externalizing psychopathology (*β* = −0.12). Greater externalizing psychopathology then increased SI (*β* = 0.17), and SI increased NSSI (*β* = 0.24). There was also a pathway where more internalizing psychopathology increased NSSI (*β* = 0.09). Similar to males, increased higher cognitive performance was protective against SITBs and internalizing psychopathology had a direct causal pathway to NSSI. There was one connection between internalizing and externalizing psychopathology such that it could either be a unidirectional path from internalizing to externalizing psychopathology (*β* = 0.60), or there is an unmeasured latent confounder such that there are no causal pathways between these two variables.

## 4 Discussion

The current study examined possible causal relationships between SI, NSSI, and relevant psychological, cognitive, and neural factors within the context of early adolescence. The use of CDA on a large observational, cross‐sectional dataset provides the opportunity to begin untangling the complexity of suicide risk specific to early adolescence. This multilevel study demonstrates the utility of CDA, in that these data were necessarily collected under circumstances where a randomized controlled trial was not implemented or feasible.

When looking at all three graphs (i.e., full sample, males, females), our initial hypothesis was largely supported. With one exception (rsFC was sometimes, but was not consistently found to have a causal path to cognitive performance), a causal pathway similar to our hypothesized top‐down structure was discovered such that better cognitive performance decreased psychopathology and psychopathology increased SI, leading ultimately to increased NSSI. The ability to access more cognitive resources, such as executive functioning, is likely helpful in engaging in adaptive emotion regulation strategies, potentially protecting young adolescents from suffering from psychopathology. Executive functioning deficits have been linked with emotion dysregulation (Wante et al. 2017), impulsivity (Ojala et al. 2022), some types of psychopathology (White et al. 2017), and SITBs (Pu et al. 2017), highlighting mechanisms by which cognitive performance may affect SITBs. This analytic approach provided additional evidence in support of this causal chain.

The finding that SI increased NSSI in all three graphs (full, male, and female models) warrants further discussion. This is consistent with models using the ideation‐to‐action framework that view NSSI as a method of bridging the gap between SI and SA (Klonsky et al. 2021; Van Orden et al. 2010). Because SA was not included in our models due to infrequent endorsement, we did not test for SA so we were not able to evaluate these potential down‐stream effects. As a result, we were not able to test if NSSI was related to increased risk of SA, or if NSSI, for as maladaptive as it is in the long run, might be used as a method of coping with SI to prevent an SA. These results are consistent with the emotional cascade model (Selby and Joiner 2013), which states that intense negative rumination, such as SI, can cause enough distress that individuals need to find a behaviorally salient method of coping with the distress, such as NSSI. Taken together, these results suggest that interventions that are focused on equipping adolescents with emotion regulation skills such as dialectical behavior therapy (Linehan 1987) and acceptance and commitment therapy (Hayes et al. 2012) can not only help adolescents manage their internalizing and externalizing symptoms, but also enhance adaptive coping mechanisms to weaken the link between SI and NSSI. At the same time, these findings are inconsistent with some research that suggests that NSSI can cause SI (Burke et al. 2016). Nevertheless, it is possible that SI and NSSI have a cyclic causal relationship where increases in one cause increases in the other, and vice versa. Our results may be capturing one phase of the cycle. Conducting longitudinal analyses using data from future time points, along with adding SA into the models, would allow us to better understand the causal relationship between SI and NSSI in the context of risk for SA.

Looking at the full sample by itself can provide insights into general patterns that are present broadly throughout the sample. For example, the full sample model identified several causal relationships between rsFC and psychopathology that were not present in the subgraphs. More specifically, DMN and frontoparietal network rsFC increased internalizing psychopathology. Previous research has implicated increased rsFC between the DMN and frontoparietal regions in research participants with depression (Sheline et al. 2010). Additionally, cingulo‐opercular and salience network rsFC increased cingulo‐opercular and cingulo‐parietal network rsFC, which decreased externalizing psychopathology, and externalizing psychopathology separately increased SI and NSSI. These networks have been associated with externalizing psychopathology (Lees et al. 2021), suggesting that disruptions in these connections may be risk factors for SITBs by indirectly and directly increasing externalizing psychopathology. Taken together, the development of brain‐based interventions for SITBs may want to consider rsFC between these networks as targets that would apply broadly across subjects. Interventions for internalizing psychopathology may benefit from targeting DMN‐frontoparietal network rsFC specifically.

Moreover, in the full sample, the model ruled out a connection between internalizing psychopathology and NSSI. While this runs counter to intuition given this edge was able to be oriented in both male and female subgraphs, this is likely due to the structure of the graph and the causal logic used to orient edges. Briefly, in the full sample graph, which combines male and female subgraphs, externalizing psychopathology, internalizing psychopathology, SI, and NSSI are interconnected to one another (i.e., forming a “clique” in graph theory). Because no causal pathways to either internalizing psychopathology or NSSI from a variable outside of this clique exist, the direction of the internalizing psychopathology–NSSI edge cannot be oriented because it is covered or obscured by their parent nodes, which are internalizing and externalizing psychopathology (Kummerfeld 2021). This suggests that analysis of the sample as a whole may also obscure certain causal relationships that are identifiable when stratifying the sample by assigned sex at birth.

Embedding these findings within early adolescence is critical. In this study, more males endorsed NSSI than females. Historically, research has focused primarily on middle‐ to late‐ adolescence where the high prevalence in females has been emphasized. Research rarely includes youth that are in late childhood or early adolescence. The results of this study highlight how both SI and NSSI at age 9–10 are overrepresented in males, a finding that must be highlighted when interpreting these results. While the current dataset did not include data on NSSI methods, it is possible that the NSSI behaviors reported at this developmental stage largely consist of behaviors that are commonly associated with externalizing symptoms. Additionally, males may choose to self‐harm in areas of the body that are more visible when compared to females (McLouth et al. 2012). Thus, it is possible that males engage in NSSI using methods that tend to overlap with externalizing behaviors, which are more easily observed and reported by parents (Barrocas et al. 2012), possibly explaining the overrepresentation of NSSI endorsement in males in the current sample. This relationship is expected to change as SITBs become more prevalent in females as the sample advances through the adolescent period (Moloney et al. 2024). Despite the large sample, the rates of endorsement of SI and NSSI were relatively low. Ideally, SA would be included in the model; however, endorsement was exceptionally rare in this age group. Future research may want to incorporate later years of ABCD data where rates of SA may be more frequent to fully model the relationship between SI, NSSI, and SA. Understanding how this relationship changes over time in males vs. females would also be an important next step.

There were several differences noted when visually comparing the male and female subgraphs. First, the female model discovered an indirect positive causal relationship between externalizing psychopathology and NSSI such that externalizing psychopathology → SI → NSSI. This relationship between externalizing psychopathology and SITBs in males was less clear, leaving open the possibility that there could be a latent confounder underlying the link between externalizing psychopathology and SI in males. Additionally, in the male subgraph, there is a direct path from cognitive performance to SI, whereas the female subgraph has an indirect path from cognitive performance to SI through externalizing psychopathology. However, in both the male and female subgraphs, internalizing psychopathology is directly linked with NSSI. While the path between internalizing psychopathology and NSSI is consistent with existing literature (Zhou et al. 2024), the link between externalizing psychopathology and SI is less well supported. There is some literature supporting the relationships between both internalizing and externalizing psychopathology with SI (Johnson et al. 2020), which indicates that SI may be a byproduct of overall self‐regulation difficulties and distress. These results suggest that there are nuanced, complex differences in the pathways to SITB risk between males and females. Existing research on adolescent SITBs tends to focus on mid‐ to late‐adolescence, when females, who are more likely to report higher levels of internalizing symptoms when compared to males (Matos et al. 2017), begin to surpass males in the endorsement of SITBs (Moloney et al. 2024). Much less is known about the mechanisms and correlates of SITBs in early adolescents. These complexities in the findings between males and females highlight the importance of research on the early adolescent period as rates of SITBs engagement, particularly NSSI, differ during this time when compared to mid‐ and late‐adolescence. Clinically, thorough assessments for SITBs should be conducted when there is elevated psychopathology, irrespective of whether or not symptoms tend to match an internalizing or externalizing profile and whether or not the symptoms are expected or socially acceptable given an adolescent's assigned sex at birth (Glatz and Buchanan 2023).

Additionally, the male model identified causal paths that were absent in the female model such that cingulo‐parietal and frontoparietal network rsFC decreased cognitive performance and cognitive performance directly decreased SI whereas cognitive performance indirectly decreased SI by decreasing externalizing psychopathology in females. There is limited evidence suggesting that there is a negative relationship between cognitive performance and connectivity in the frontoparietal and cingulo‐parietal networks (Hearne et al. 2016; Wagner et al. 2010); therefore, this result from the male graph should be considered preliminary. Taken together, these findings suggest that there may be subtle but important differences in the risk and protective processes for SITBs between males and females.

The current study used between‐network rsFC as the measure of neural activity. There are many potential relevant brain indexes that could have been considered. Previous research has linked brain activation and functional connectivity during tasks to SITBs (Wiglesworth et al. 2023). Future studies may benefit from exploring other brain‐based measures and their relationships to SITBs. Also, while the current study included measures from multiple domains, the analyses did not include commonly used sociodemographic and behavioral measures (e.g., impulsivity) in the interest of reducing required computing power through reducing model complexity. While GFCI, the CDA algorithm chosen for this study, does not require causal sufficiency (i.e., all confounding variables are observed and included in the model; Ogarrio et al. 2016), nevertheless, inclusion of additional relevant factors would likely serve to increase the comprehensiveness of the models and thus our understanding of the mechanisms of SITBs (e.g., the identification of additional, unique risk pathways in the model). For example, there are documented mental health impacts of various physiological (e.g., sleep; Tarokh et al. 2016), social (e.g., peer victimization; Oncioiu et al. 2023), and environmental (e.g., neighborhood safety; Min et al. 2024) factors. These variables may provide more information on how mechanisms of risk for SITBs operate in different contexts. Future studies should incorporate these measures to further clarify the observed results. Finally, it is unclear to what extent reporting biases on suicide assessments impacted the observed results. Prior research has found that there is inconsistent reporting of SITBs over time in the ABCD Study (Wiglesworth et al. 2025). As such, it is difficult to determine the accuracy of these SITB assessments. More research is needed to establish an optimal method of assessing suicide risk, particularly in young adolescents.

## 5 Conclusion

The CDA models suggest direct and indirect causal pathways of SITBs, including evidence that alterations in functional connectivity, cognitive performance, externalizing symptoms and can increase suicide risk in adolescents at this age. Additionally, causal pathways between SI and NSSI were identified, but suggest that comprehensive assessments for NSSI and SI risk should take into account assigned sex at birth for this age group.

## Ethics Statement

This study consisted of secondary analyses of data collected from the baseline visit of the Adolescent Brain Cognitive Development (ABCD) Study. Each ABCD data collection site obtained institutional review board (IRB) approval either locally or from a central IRB at the University of California San Diego.

## Consent

Informed consent and assent was obtained from parents and adolescents at their respective site.

## Conflicts of Interest

The authors declare no conflicts of interest.

## Supporting information


**Data S1:** sltb70068‐sup‐0001‐Supinfo.docx.

## Data Availability

The data that support the findings of this study are openly available in the ABCD Data Repository at http://doi.org/10.15154/z563‐zd24.
