# Supplementary material for: Examining Causal Pathways to Suicidal Ideation and Nonsuicidal Self‐Injury in the Adolescent Brain Cognitive Development Study
Source: Suicide Life Threat Behav. 2025 Nov 24;55(6):e70068. doi: 10.1111/sltb.70068 (PMC12641592; doi:10.1111/sltb.70068)
Supplement: Supplementary file 1 — Data S1: sltb70068‐sup‐0001‐Supinfo.docx. [file SLTB-55-0-s001.docx]

**Supporting Information**

**Supplementary Methods**

***Psychopathology***

The Child Behavior Checklist (CBCL) is a 113 item parent-reported measure on behaviors observed in their child [(Achenbach, 1999)](https://www.zotero.org/google-docs/?RDNgsg). The CBCL has eight syndrome scales: aggressive behavior, anxious/depressed, attention problems, rule-breaking behavior, somatic complaints, social problems, thought problems, and withdrawn/depressed. The current study utilized the internalizing (combines anxious/depressed, withdrawn/depressed, and somatic complaints syndrome scales) and externalizing (combines rule-breaking and aggressive behavior syndrome scales) psychopathology subscales. The current study used the standardized scores (i.e., t-scores) for the internalizing and externalizing subscales which adjusts the raw scores based on the age of the participant.

***Neurocognition***

The current study utilized the age-corrected standard score for the NIH Toolbox cognitive function composite, which was calculated by averaging the fluid (i.e., scores from the Flanker Inhibitory Control & Attention, Picture Sequence Memory, Dimensional Change Card Sort, Pattern Comparison Processing Speed, and List Sorting Working Memory tasks) and crystallized (i.e., scores from the Picture Vocabulary and Oral Reading Recognition tasks) composite standard scores. The age-correction process compared an individual’s scores to those from a representative sample acquired by NIH Toolbox. Thus, the age-corrected standard cognitive function composite score used in the current study represents an individual’s general cognitive abilities relative to peers their age.

***Neuroimaging***

Neuroimaging data from the resting-state functional magnetic resonance imaging (rs-fMRI) scans were included in the current study. ABCD publishes a set of recommended criteria for inclusion for all their imaging data [(Jernigan et al., 2024)](https://www.zotero.org/google-docs/?yKrThU). We used the guidelines for rs-fMRI data, which included common practices for preparing neuroimaging data such as multiple quality control checks, successful registration of the rs-fMRI data to the structural T1 scans, and making sure key regions were not cut off during image acquisition.

***Casual Discovery Analysis***

The greedy fast causal inference (GFCI) algorithm [(Ogarrio et al., 2016)](https://www.zotero.org/google-docs/?dEQylM) starts with an empty graph and uses two other algorithms to discover a causal graph. The first step uses the fast greedy equivalence search (FGES)[(Ramsey et al., 2017)](https://www.zotero.org/google-docs/?KdwUGM) algorithm to add edges between variables to improve an index of model fit. In the current study, the bayesian information criterion (BIC)[(Schwarz, 1978)](https://www.zotero.org/google-docs/?hwmhAR) was used as the measures of model fit. Once the graph is in a state such that adding an edge would no longer improve the BIC, FGES starts to remove edges that will improve the BIC until removing edges will also no longer improve the BIC. Next, GFCI uses the fast causal inference (FCI)[(Spirtes et al., 2001)](https://www.zotero.org/google-docs/?6VJNoW) algorithm to orient the directions of the edges based on a set of causal assumptions [(Glymour et al., 2019)](https://www.zotero.org/google-docs/?XISP2O). Of note, GFCI was chosen specifically due to its relaxation of the causal sufficiency assumption [(Ogarrio et al., 2016)](https://www.zotero.org/google-docs/?GUQqNm).

Supplementary References

[Achenbach, T. M. (1999). The Child Behavior Checklist and related instruments. In *The use of psychological testing for treatment planning and outcomes assessment, 2nd ed* (pp. 429–466). Lawrence Erlbaum Associates Publishers.](https://www.zotero.org/google-docs/?AgIIAb)

[Gershon, R. C., Wagster, M. V., Hendrie, H. C., Fox, N. A., Cook, K. F., & Nowinski, C. J. (2013). NIH Toolbox for Assessment of Neurological and Behavioral Function. *Neurology*, *80*(11 Suppl 3), S2–S6. https://doi.org/10.1212/WNL.0b013e3182872e5f](https://www.zotero.org/google-docs/?AgIIAb)

[Glymour, C., Zhang, K., & Spirtes, P. (2019). Review of Causal Discovery Methods Based on Graphical Models. *Frontiers in Genetics*, *10*. https://doi.org/10.3389/fgene.2019.00524](https://www.zotero.org/google-docs/?AgIIAb)

[Jernigan, T. L., Brown, S. A., Dale, A. M., Tapert, S., Sowell, E. R., Herting, M., Laird, A., Gonzalez, R., Squeglia, L. M., Gray, K., Paulus, M. P., Aupperle, R., Feldstein Ewing, S. W., Nagel, B. J., Fair, D. A., Baker, F., Colrain, I. M., Bookheimer, S. Y., Dapretto, M., … Gee, D. (2024). *Adolescent Brain Cognitive Development Study (ABCD)—Annual Release 5.1 #2313*. https://doi.org/10.15154/z563-zd24](https://www.zotero.org/google-docs/?AgIIAb)

[Ogarrio, J. M., Spirtes, P., & Ramsey, J. (2016). A Hybrid Causal Search Algorithm for Latent Variable Models. *JMLR Workshop and Conference Proceedings*, *52*, 368–379.](https://www.zotero.org/google-docs/?AgIIAb)

[Ramsey, J., Glymour, M., Sanchez-Romero, R., & Glymour, C. (2017). A million variables and more: The Fast Greedy Equivalence Search algorithm for learning high-dimensional graphical causal models, with an application to functional magnetic resonance images. *International Journal of Data Science and Analytics*, *3*(2), 121–129. https://doi.org/10.1007/s41060-016-0032-z](https://www.zotero.org/google-docs/?AgIIAb)

[Schwarz, G. (1978). Estimating the Dimension of a Model. *The Annals of Statistics*, *6*(2), 461–464. https://doi.org/10.1214/aos/1176344136](https://www.zotero.org/google-docs/?AgIIAb)

[Spirtes, P., Glymour, C., & Scheines, R. (2001). *Causation, Prediction, and Search* (Second Edition). The MIT Press. https://direct.mit.edu/books/monograph/2057/Causation-Prediction-and-Search](https://www.zotero.org/google-docs/?AgIIAb)

**Supplementary Tables**

**Table S1.** Confidence Intervals for Pathways in Full Sample Graph

| **Node 1** | **Edge Type** | **Node 2** | **Standard Estimate** | **Standard Error** | **95% Lower Confidence Interval** | **95% Upper Confidence Interval** |
| --- | --- | --- | --- | --- | --- | --- |
| NSSI | ~ | SI | 0.199017 | 0.010442 | 0.178551 | 0.219483 |
| NSSI | ~ | Externalizing | 0.089372 | 0.01045 | 0.068891 | 0.109854 |
| SI | ~ | Externalizing | 0.16649 | 0.010491 | 0.145928 | 0.187052 |
| SI | ~ | Cognitive Performance | -0.04203 | 0.010461 | -0.06254 | -0.02153 |
| Externalizing | ~ | Cognitive Performance | -0.11412 | 0.008475 | -0.13073 | -0.09751 |
| Externalizing | ~ | Cingulo-opercular- Cingulo-parietal RSFC | -0.03232 | 0.008477 | -0.04893 | -0.0157 |
| Cingulo-parietal - Salience RSFC | ~ | Cognitive Performance | -0.04817 | 0.010471 | -0.06869 | -0.02765 |
| Cingulo-parietal - Default Mode RSFC | ~ | Cingulo-parietal - Frontoparietal RSFC | 0.113036 | 0.009291 | 0.094826 | 0.131246 |
| Cingulo-parietal - Default Mode RSFC | ~ | Cingulo-parietal - Salience RSFC | 0.05145 | 0.010933 | 0.030021 | 0.072879 |
| Cingulo-opercular- Cingulo-parietal RSFC | ~ | Cingulo-opercular - Salience RSFC | 0.08112 | 0.008084 | 0.065277 | 0.096964 |
| Internalizing | ~ | Default Mode - Frontoparietal RSFC | 0.018646 | 0.008495 | 0.001997 | 0.035295 |
| Cingulo-opercular- Default Mode RSFC | ~ | Default Mode - Frontoparietal RSFC | 0.128893 | 0.010471 | 0.108369 | 0.149416 |
| NSSI | ~~ | Internalizing | 0.057561 | 0.0083 | 0.041294 | 0.073828 |
| SI | ~~ | Internalizing | 0.062074 | 0.008427 | 0.045557 | 0.078591 |
| Externalizing | ~~ | Internalizing | 0.584871 | 0.012175 | 0.561008 | 0.608734 |
| Cognitive Performance | ~~ | Cingulo-parietal - Frontoparietal RSFC | -0.06598 | 0.010393 | -0.08636 | -0.04561 |
| Cingulo-opercular- Cingulo-parietal RSFC | ~~ | Cognitive Performance | 0.051831 | 0.008766 | 0.03465 | 0.069012 |
| Cognitive Performance | ~~ | Cingulo-opercular - Frontoparietal RSFC | -0.05518 | 0.008455 | -0.07175 | -0.03861 |
| Cognitive Performance | ~~ | Default Mode-Salience RSFC | 0.06182 | 0.009046 | 0.044089 | 0.07955 |
| Cingulo-parietal - Default Mode RSFC | ~~ | Default Mode-Salience RSFC | 0.120585 | 0.007565 | 0.105757 | 0.135412 |
| Cingulo-parietal - Frontoparietal RSFC | ~~ | Frontoparietal-Salience RSFC | 0.156815 | 0.009843 | 0.137523 | 0.176106 |
| Cingulo-parietal - Salience RSFC | ~~ | Cingulo-parietal - Frontoparietal RSFC | 0.303566 | 0.010481 | 0.283024 | 0.324109 |
| Cingulo-parietal - Salience RSFC | ~~ | Frontoparietal-Salience RSFC | 0.101237 | 0.00904 | 0.083518 | 0.118956 |
| Cingulo-parietal - Default Mode RSFC | ~~ | Cingulo-opercular- Cingulo-parietal RSFC | -0.53915 | 0.01128 | -0.56126 | -0.51704 |
| Cingulo-parietal - Salience RSFC | ~~ | Cingulo-opercular- Cingulo-parietal RSFC | 0.312989 | 0.010483 | 0.292444 | 0.333535 |
| Cingulo-opercular- Default Mode RSFC | ~~ | Cingulo-opercular - Frontoparietal RSFC | 0.35871 | 0.009572 | 0.339949 | 0.37747 |
| Cingulo-opercular- Default Mode RSFC | ~~ | Default Mode-Salience RSFC | 0.22222 | 0.008934 | 0.20471 | 0.239729 |
| Cingulo-opercular - Salience RSFC | ~~ | Cingulo-opercular - Frontoparietal RSFC | 0.184645 | 0.008872 | 0.167256 | 0.202034 |
| Default Mode - Frontoparietal RSFC | ~~ | Cingulo-opercular - Frontoparietal RSFC | -0.29058 | 0.010374 | -0.31091 | -0.27025 |
| Cingulo-opercular - Frontoparietal RSFC | ~~ | Frontoparietal-Salience RSFC | 0.227769 | 0.009843 | 0.208477 | 0.247062 |
| Cingulo-opercular - Salience RSFC | ~~ | Default Mode-Salience RSFC | -0.37689 | 0.010509 | -0.39749 | -0.35629 |
| Cingulo-opercular - Salience RSFC | ~~ | Frontoparietal-Salience RSFC | -0.04935 | 0.009513 | -0.06799 | -0.0307 |
| Default Mode - Frontoparietal RSFC | ~~ | Default Mode-Salience RSFC | 0.274351 | 0.009628 | 0.255481 | 0.293221 |
| Default Mode - Frontoparietal RSFC | ~~ | Frontoparietal-Salience RSFC | 0.169131 | 0.010146 | 0.149245 | 0.189018 |
| NSSI | ~~ | NSSI | 0.945842 | 0.014149 | 0.91811 | 0.973574 |
| SI | ~~ | SI | 0.968553 | 0.014489 | 0.940155 | 0.996951 |
| Internalizing | ~~ | Internalizing | 0.999091 | 0.014946 | 0.969798 | 1.028384 |
| Externalizing | ~~ | Externalizing | 0.983648 | 0.014715 | 0.954807 | 1.012489 |
| Cognitive Performance | ~~ | Cognitive Performance | 0.999814 | 0.014955 | 0.970503 | 1.029126 |
| Cingulo-parietal - Frontoparietal RSFC | ~~ | Cingulo-parietal - Frontoparietal RSFC | 0.999545 | 0.014951 | 0.970242 | 1.028849 |
| Cingulo-opercular- Cingulo-parietal RSFC | ~~ | Cingulo-opercular- Cingulo-parietal RSFC | 0.990519 | 0.014798 | 0.961516 | 1.019522 |
| Cingulo-opercular - Frontoparietal RSFC | ~~ | Cingulo-opercular - Frontoparietal RSFC | 0.976071 | 0.014276 | 0.94809 | 1.004052 |
| Default Mode-Salience RSFC | ~~ | Default Mode-Salience RSFC | 1.022248 | 0.014936 | 0.992975 | 1.051522 |
| Cingulo-parietal - Default Mode RSFC | ~~ | Cingulo-parietal - Default Mode RSFC | 0.984105 | 0.014619 | 0.955452 | 1.012759 |
| Frontoparietal-Salience RSFC | ~~ | Frontoparietal-Salience RSFC | 0.999405 | 0.014883 | 0.970234 | 1.028576 |
| Cingulo-parietal - Salience RSFC | ~~ | Cingulo-parietal - Salience RSFC | 0.993069 | 0.01471 | 0.964237 | 1.021901 |
| Cingulo-opercular- Default Mode RSFC | ~~ | Cingulo-opercular- Default Mode RSFC | 0.980995 | 0.014562 | 0.952455 | 1.009536 |
| Cingulo-opercular - Salience RSFC | ~~ | Cingulo-opercular - Salience RSFC | 1.000414 | 0.014866 | 0.971277 | 1.029551 |
| Default Mode - Frontoparietal RSFC | ~~ | Default Mode - Frontoparietal RSFC | 0.998403 | 0.014757 | 0.969479 | 1.027327 |
| NSSI | ~~ | Cingulo-parietal - Default Mode RSFC | 0.00016 | 0.008201 | -0.01591 | 0.016234 |
| NSSI | ~~ | Cingulo-opercular- Default Mode RSFC | 0.01539 | 0.009127 | -0.0025 | 0.033278 |
| Cingulo-parietal - Default Mode RSFC | ~~ | Internalizing | 4.16E-05 | 0.006785 | -0.01326 | 0.013339 |
| Cingulo-parietal - Default Mode RSFC | ~~ | Cingulo-opercular- Default Mode RSFC | -0.00953 | 0.007719 | -0.02466 | 0.005602 |
| Internalizing | ~~ | Cingulo-opercular- Default Mode RSFC | -0.00272 | 0.00755 | -0.01752 | 0.012076 |

**Table S1.** An edge type of “~” denotes a unidirectional causal pathway from Node 2 to Node 1. An edge type of “~~” denotes that there are no causal pathways between Node 1 and Node 2 because unmeasured latent confounder(s) were discovered. When there is a “~~” between the same variable in Node 1 and Node 2, that represents the variance of that variable.

**Table S2.** Confidence Intervals for Pathways in Male Subsample Graph

| **Node 1** | **Edge Type** | **Node 2** | **Standard Estimate** | **Standard Error** | **95% Lower Confidence Interval** | **95% Upper Confidence Interval** |
| --- | --- | --- | --- | --- | --- | --- |
| NSSI | ~ | SI | 0.157472 | 0.014781 | 0.128502 | 0.186441 |
| NSSI | ~ | Internalizing | 0.120266 | 0.014777 | 0.091303 | 0.149229 |
| SI | ~ | Externalizing | 0.095609 | 0.018026 | 0.060279 | 0.130938 |
| SI | ~ | Cognitive Performance | -0.05147 | 0.014729 | -0.08034 | -0.02261 |
| Externalizing | ~ | Internalizing | 0.574293 | 0.012064 | 0.550648 | 0.597938 |
| Externalizing | ~ | Cognitive Performance | -0.11011 | 0.012066 | -0.13376 | -0.08647 |
| Cognitive Performance | ~ | Cingulo-parietal - Frontoparietal RSFC | -0.08194 | 0.014793 | -0.11094 | -0.05295 |
| Frontoparietal-Salience RSFC | ~ | Cingulo-parietal - Frontoparietal RSFC | 0.192658 | 0.013358 | 0.166477 | 0.218839 |
| Frontoparietal-Salience RSFC | ~ | Cingulo-opercular - Salience RSFC | -0.08089 | 0.0142 | -0.10872 | -0.05306 |
| Frontoparietal-Salience RSFC | ~ | Default Mode - Frontoparietal RSFC | 0.163845 | 0.014208 | 0.135997 | 0.191693 |
| Cingulo-parietal - Default Mode RSFC | ~ | Cingulo-parietal - Salience RSFC | 0.092072 | 0.014468 | 0.063716 | 0.120429 |
| Cingulo-parietal - Default Mode RSFC | ~ | Default Mode-Salience RSFC | 0.173806 | 0.012325 | 0.149649 | 0.197964 |
| Cingulo-opercular- Cingulo-parietal RSFC | ~ | Cingulo-opercular - Salience RSFC | 0.107862 | 0.011784 | 0.084766 | 0.130957 |
| Cingulo-opercular- Default Mode RSFC | ~ | Cingulo-opercular - Salience RSFC | 0.163489 | 0.014588 | 0.134897 | 0.192081 |
| SI | ~~ | Internalizing | 0.120661 | 0.018067 | 0.085249 | 0.156072 |
| Cognitive Performance | ~~ | Cingulo-opercular - Frontoparietal RSFC | -0.07447 | 0.011869 | -0.09773 | -0.0512 |
| Cingulo-parietal - Default Mode RSFC | ~~ | Cingulo-parietal - Frontoparietal RSFC | 0.093721 | 0.011934 | 0.070332 | 0.11711 |
| Cingulo-parietal - Frontoparietal RSFC | ~~ | Cingulo-parietal - Salience RSFC | 0.314793 | 0.014914 | 0.285563 | 0.344023 |
| Cingulo-parietal - Default Mode RSFC | ~~ | Cingulo-opercular- Cingulo-parietal RSFC | -0.52191 | 0.01572 | -0.55272 | -0.4911 |
| Cingulo-opercular- Cingulo-parietal RSFC | ~~ | Cingulo-parietal - Salience RSFC | 0.305321 | 0.014761 | 0.276391 | 0.334251 |
| Cingulo-opercular- Default Mode RSFC | ~~ | Cingulo-parietal - Salience RSFC | -0.03866 | 0.012566 | -0.06329 | -0.01403 |
| Cingulo-opercular- Default Mode RSFC | ~~ | Cingulo-opercular - Frontoparietal RSFC | 0.347744 | 0.014028 | 0.320251 | 0.375238 |
| Cingulo-opercular- Default Mode RSFC | ~~ | Default Mode-Salience RSFC | 0.220891 | 0.012654 | 0.196089 | 0.245692 |
| Cingulo-opercular - Salience RSFC | ~~ | Cingulo-opercular - Frontoparietal RSFC | 0.248864 | 0.01417 | 0.221092 | 0.276637 |
| Default Mode - Frontoparietal RSFC | ~~ | Cingulo-opercular - Frontoparietal RSFC | -0.33463 | 0.014125 | -0.36231 | -0.30694 |
| Frontoparietal-Salience RSFC | ~~ | Cingulo-opercular - Frontoparietal RSFC | 0.301621 | 0.013721 | 0.274728 | 0.328513 |
| Cingulo-opercular - Salience RSFC | ~~ | Default Mode-Salience RSFC | -0.32022 | 0.014706 | -0.34904 | -0.2914 |
| Default Mode - Frontoparietal RSFC | ~~ | Default Mode-Salience RSFC | 0.280206 | 0.013593 | 0.253563 | 0.306848 |
| NSSI | ~~ | NSSI | 0.953525 | 0.020095 | 0.914138 | 0.992911 |
| SI | ~~ | SI | 0.973215 | 0.020662 | 0.932719 | 1.013712 |
| Externalizing | ~~ | Externalizing | 0.655211 | 0.013808 | 0.628146 | 0.682275 |
| Cognitive Performance | ~~ | Cognitive Performance | 0.992758 | 0.020922 | 0.951751 | 1.033764 |
| Frontoparietal-Salience RSFC | ~~ | Frontoparietal-Salience RSFC | 0.922406 | 0.019439 | 0.884308 | 0.960505 |
| Cingulo-parietal - Default Mode RSFC | ~~ | Cingulo-parietal - Default Mode RSFC | 0.960806 | 0.020196 | 0.921224 | 1.000389 |
| Cingulo-opercular- Cingulo-parietal RSFC | ~~ | Cingulo-opercular- Cingulo-parietal RSFC | 0.986942 | 0.020799 | 0.946177 | 1.027707 |
| Cingulo-opercular- Default Mode RSFC | ~~ | Cingulo-opercular- Default Mode RSFC | 0.967939 | 0.020254 | 0.928241 | 1.007637 |
| Internalizing | ~~ | Internalizing | 0.999778 | 0.02107 | 0.958482 | 1.041075 |
| Cingulo-parietal - Frontoparietal RSFC | ~~ | Cingulo-parietal - Frontoparietal RSFC | 0.999778 | 0.02107 | 0.958482 | 1.041075 |
| Cingulo-opercular - Salience RSFC | ~~ | Cingulo-opercular - Salience RSFC | 1.004235 | 0.021017 | 0.963043 | 1.045427 |
| Default Mode - Frontoparietal RSFC | ~~ | Default Mode - Frontoparietal RSFC | 1.005021 | 0.020976 | 0.963908 | 1.046135 |
| Cingulo-parietal - Salience RSFC | ~~ | Cingulo-parietal - Salience RSFC | 0.999075 | 0.020853 | 0.958205 | 1.039946 |
| Default Mode-Salience RSFC | ~~ | Default Mode-Salience RSFC | 0.988186 | 0.020565 | 0.947879 | 1.028493 |
| Cingulo-opercular - Frontoparietal RSFC | ~~ | Cingulo-opercular - Frontoparietal RSFC | 1.036397 | 0.021292 | 0.994666 | 1.078128 |
| NSSI | ~~ | Frontoparietal-Salience RSFC | -0.0117 | 0.01311 | -0.0374 | 0.013994 |
| NSSI | ~~ | Cingulo-parietal - Default Mode RSFC | 0.012901 | 0.014192 | -0.01492 | 0.040718 |
| NSSI | ~~ | Cingulo-opercular- Cingulo-parietal RSFC | -0.01911 | 0.01368 | -0.04592 | 0.0077 |
| NSSI | ~~ | Cingulo-opercular- Default Mode RSFC | 0.012284 | 0.012983 | -0.01316 | 0.03773 |
| Frontoparietal-Salience RSFC | ~~ | Cingulo-parietal - Default Mode RSFC | -0.01376 | 0.013094 | -0.03942 | 0.011908 |
| Frontoparietal-Salience RSFC | ~~ | Cingulo-opercular- Cingulo-parietal RSFC | 0.001004 | 0.012618 | -0.02373 | 0.025735 |
| Frontoparietal-Salience RSFC | ~~ | Cingulo-opercular- Default Mode RSFC | 0.025444 | 0.013627 | -0.00126 | 0.052151 |
| Cingulo-parietal - Default Mode RSFC | ~~ | Cingulo-opercular- Default Mode RSFC | -0.01613 | 0.012975 | -0.04156 | 0.009301 |
| Cingulo-opercular- Cingulo-parietal RSFC | ~~ | Cingulo-opercular- Default Mode RSFC | 0.002292 | 0.013213 | -0.0236 | 0.028189 |

**Table S2.** An edge type of “~” denotes a unidirectional causal pathway from Node 2 to Node 1. An edge type of “~~” denotes that there are no causal pathways between Node 1 and Node 2 because unmeasured latent confounder(s) were discovered. When there is a “~~” between the same variable in Node 1 and Node 2, that represents the variance of that variable.

**Table S3.** Confidence Intervals for Pathways in Female Subsample Graph

| **Node 1** | **Edge Type** | **Node 2** | **Standard Estimate** | **Standard Error** | **95% Lower Confidence Interval** | **95% Upper Confidence Interval** |
| --- | --- | --- | --- | --- | --- | --- |
| NSSI | ~ | SI | 0.249983 | 0.014488 | 0.221588 | 0.278378 |
| NSSI | ~ | Internalizing | 0.093464 | 0.014487 | 0.06507 | 0.121859 |
| SI | ~ | Externalizing | 0.169621 | 0.01481 | 0.140594 | 0.198647 |
| Externalizing | ~ | Internalizing | 0.593277 | 0.011939 | 0.569876 | 0.616677 |
| Externalizing | ~ | Cognitive Performance | -0.12105 | 0.011939 | -0.14445 | -0.09765 |
| Cingulo-parietal - Frontoparietal RSFC | ~ | Cognitive Performance | -0.06507 | 0.014835 | -0.09415 | -0.036 |
| Cingulo-parietal - Salience RSFC | ~ | Cingulo-parietal - Default Mode RSFC | 0.060314 | 0.014312 | 0.032263 | 0.088364 |
| Cingulo-parietal - Salience RSFC | ~ | Cingulo-parietal - Frontoparietal RSFC | 0.30615 | 0.014263 | 0.278196 | 0.334105 |
| Cingulo-opercular- Cingulo-parietal RSFC | ~ | Cingulo-parietal - Salience RSFC | 0.368218 | 0.011437 | 0.345801 | 0.390635 |
| Cingulo-parietal - Default Mode RSFC | ~ | Cingulo-parietal - Frontoparietal RSFC | 0.068383 | 0.012014 | 0.044835 | 0.09193 |
| Cingulo-opercular- Default Mode RSFC | ~ | Default Mode - Frontoparietal RSFC | 0.118409 | 0.014797 | 0.089408 | 0.14741 |
| Frontoparietal-Salience RSFC | ~ | Default Mode - Frontoparietal RSFC | 0.180559 | 0.014521 | 0.152098 | 0.20902 |
| Cingulo-opercular- Cingulo-parietal RSFC | ~~ | Cognitive Performance | 0.071867 | 0.011354 | 0.049613 | 0.09412 |
| Cognitive Performance | ~~ | Default Mode-Salience RSFC | 0.076484 | 0.01271 | 0.051573 | 0.101395 |
| Cingulo-parietal - Default Mode RSFC | ~~ | Default Mode-Salience RSFC | 0.099259 | 0.010742 | 0.078204 | 0.120313 |
| Cingulo-parietal - Frontoparietal RSFC | ~~ | Frontoparietal-Salience RSFC | 0.133203 | 0.013947 | 0.105867 | 0.160538 |
| Cingulo-opercular- Cingulo-parietal RSFC | ~~ | Cingulo-parietal - Default Mode RSFC | -0.55965 | 0.016299 | -0.5916 | -0.52771 |
| Cingulo-opercular- Cingulo-parietal RSFC | ~~ | Cingulo-opercular - Salience RSFC | 0.06326 | 0.010357 | 0.04296 | 0.08356 |
| Cingulo-opercular- Default Mode RSFC | ~~ | Cingulo-opercular - Frontoparietal RSFC | 0.381664 | 0.0145 | 0.353246 | 0.410083 |
| Cingulo-opercular- Default Mode RSFC | ~~ | Default Mode-Salience RSFC | 0.224614 | 0.012412 | 0.200287 | 0.248941 |
| Cingulo-opercular - Frontoparietal RSFC | ~~ | Cingulo-opercular - Salience RSFC | 0.18775 | 0.011785 | 0.164652 | 0.210847 |
| Default Mode - Frontoparietal RSFC | ~~ | Cingulo-opercular - Frontoparietal RSFC | -0.29847 | 0.014956 | -0.32778 | -0.26915 |
| Frontoparietal-Salience RSFC | ~~ | Cingulo-opercular - Frontoparietal RSFC | 0.303814 | 0.014164 | 0.276052 | 0.331575 |
| Cingulo-opercular - Salience RSFC | ~~ | Default Mode-Salience RSFC | -0.41011 | 0.01517 | -0.43985 | -0.38038 |
| Default Mode - Frontoparietal RSFC | ~~ | Default Mode-Salience RSFC | 0.271792 | 0.01385 | 0.244646 | 0.298938 |
| NSSI | ~~ | NSSI | 0.921891 | 0.019579 | 0.883516 | 0.960266 |
| SI | ~~ | SI | 0.97101 | 0.020622 | 0.930591 | 1.011429 |
| Externalizing | ~~ | Externalizing | 0.631902 | 0.01342 | 0.605599 | 0.658206 |
| Cingulo-parietal - Frontoparietal RSFC | ~~ | Cingulo-parietal - Frontoparietal RSFC | 0.99655 | 0.021165 | 0.955068 | 1.038033 |
| Cingulo-parietal - Salience RSFC | ~~ | Cingulo-parietal - Salience RSFC | 0.898429 | 0.019081 | 0.861031 | 0.935827 |
| Cingulo-opercular- Cingulo-parietal RSFC | ~~ | Cingulo-opercular- Cingulo-parietal RSFC | 0.891352 | 0.018861 | 0.854385 | 0.92832 |
| Cingulo-parietal - Default Mode RSFC | ~~ | Cingulo-parietal - Default Mode RSFC | 0.989233 | 0.020923 | 0.948224 | 1.030241 |
| Cingulo-opercular- Default Mode RSFC | ~~ | Cingulo-opercular- Default Mode RSFC | 0.978557 | 0.020606 | 0.93817 | 1.018943 |
| Frontoparietal-Salience RSFC | ~~ | Frontoparietal-Salience RSFC | 0.9662 | 0.02047 | 0.926079 | 1.006321 |
| Cognitive Performance | ~~ | Cognitive Performance | 0.999813 | 0.021233 | 0.958197 | 1.04143 |
| Default Mode - Frontoparietal RSFC | ~~ | Default Mode - Frontoparietal RSFC | 1.004545 | 0.021174 | 0.963044 | 1.046046 |
| Cingulo-opercular - Frontoparietal RSFC | ~~ | Cingulo-opercular - Frontoparietal RSFC | 0.999159 | 0.020904 | 0.958188 | 1.040129 |
| Cingulo-opercular - Salience RSFC | ~~ | Cingulo-opercular - Salience RSFC | 1.000895 | 0.021083 | 0.959572 | 1.042217 |
| Default Mode-Salience RSFC | ~~ | Default Mode-Salience RSFC | 1.026289 | 0.021231 | 0.984678 | 1.0679 |
| NSSI | ~~ | Cingulo-opercular- Cingulo-parietal RSFC | 0.008983 | 0.010763 | -0.01211 | 0.030077 |
| NSSI | ~~ | Cingulo-opercular- Default Mode RSFC | 0.017754 | 0.012732 | -0.0072 | 0.042708 |
| NSSI | ~~ | Frontoparietal-Salience RSFC | -0.01951 | 0.01321 | -0.0454 | 0.006381 |
| Cingulo-opercular- Cingulo-parietal RSFC | ~~ | Cingulo-opercular- Default Mode RSFC | 0.027873 | 0.010058 | 0.00816 | 0.047586 |
| Cingulo-opercular- Cingulo-parietal RSFC | ~~ | Frontoparietal-Salience RSFC | -0.02634 | 0.010331 | -0.04658 | -0.00609 |
| Cingulo-opercular- Default Mode RSFC | ~~ | Frontoparietal-Salience RSFC | 0.03998 | 0.013986 | 0.012568 | 0.067392 |
| Internalizing | ~~ | Internalizing | 0.999774 | 0 | 0.999774 | 0.999774 |

**Table S3.** An edge type of “~” denotes a unidirectional causal pathway from Node 2 to Node 1. An edge type of “~~” denotes that there are no causal pathways between Node 1 and Node 2 because unmeasured latent confounder(s) were discovered. When there is a “~~” between the same variable in Node 1 and Node 2, that represents the variance of that variable.

**Supplementary Figures**

**Figure S1.** Full Causal Graph for the Full Sample Using Lifetime SITBs
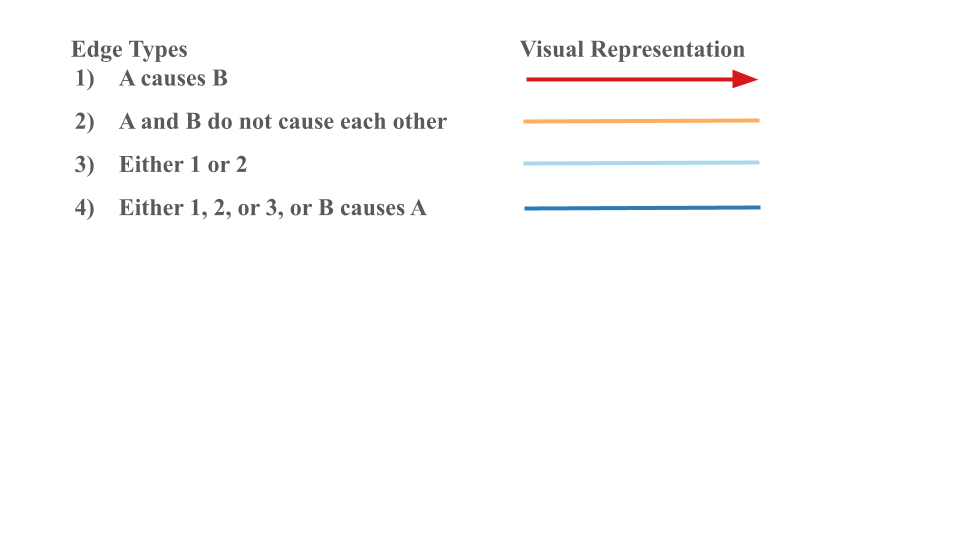


**
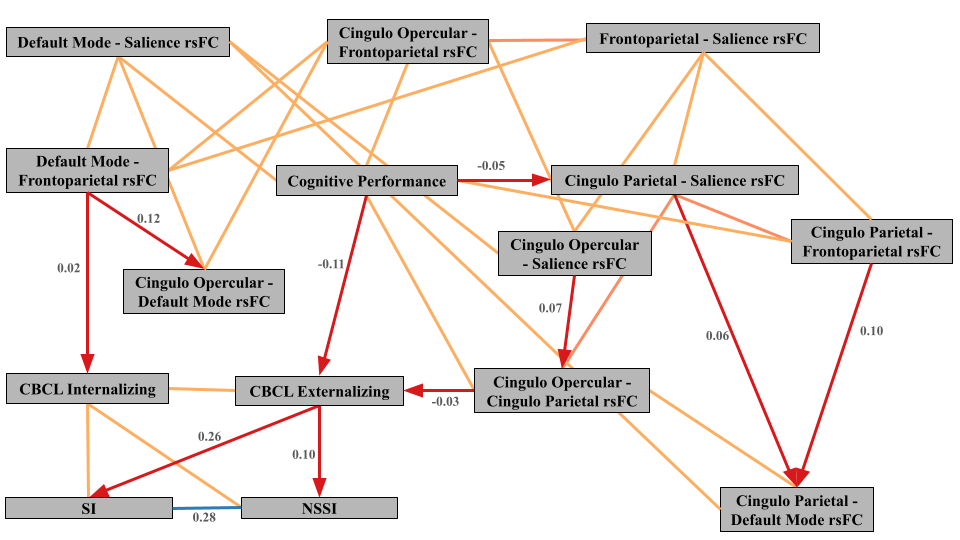
**

**Figure S1.**  Red arrows indicate a directional causal relationship was identified, orange lines indicate that the two variables did not cause each other because at least one latent confounding variable was discovered, light blue lines indicate that either a causal relationship exists in one direction or a latent confounding variable was discovered, and dark blue lines indicate that the relationship between the two variables is unclear (i.e., there could be a directional causal relationship in either direction or there could be a latent confounding variable). Standardized edge weights calculated using structural equation modeling are displayed next to each edge. All edges were significant at the *p*=.01 level.

**Figure S2.** Full Causal Graph for the Male Sample Using Lifetime SITBs
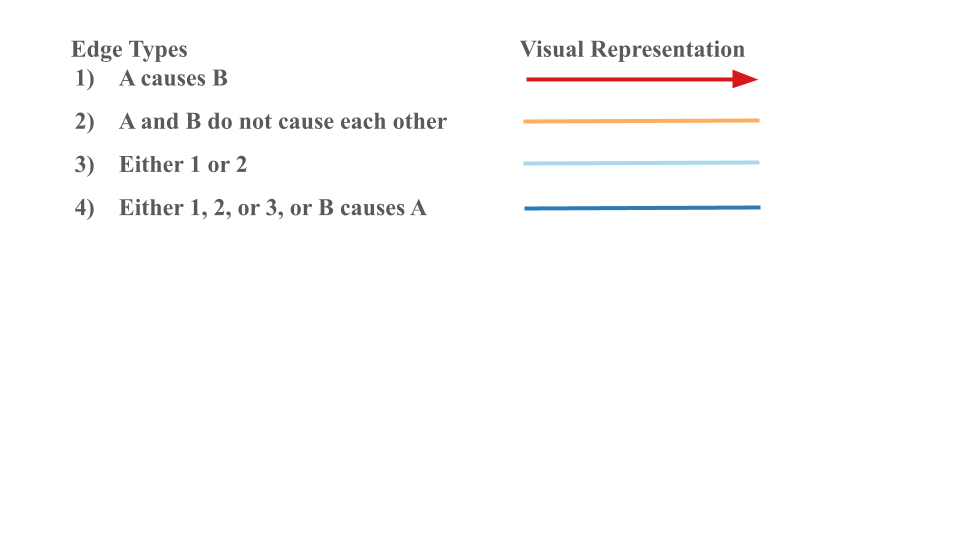


**
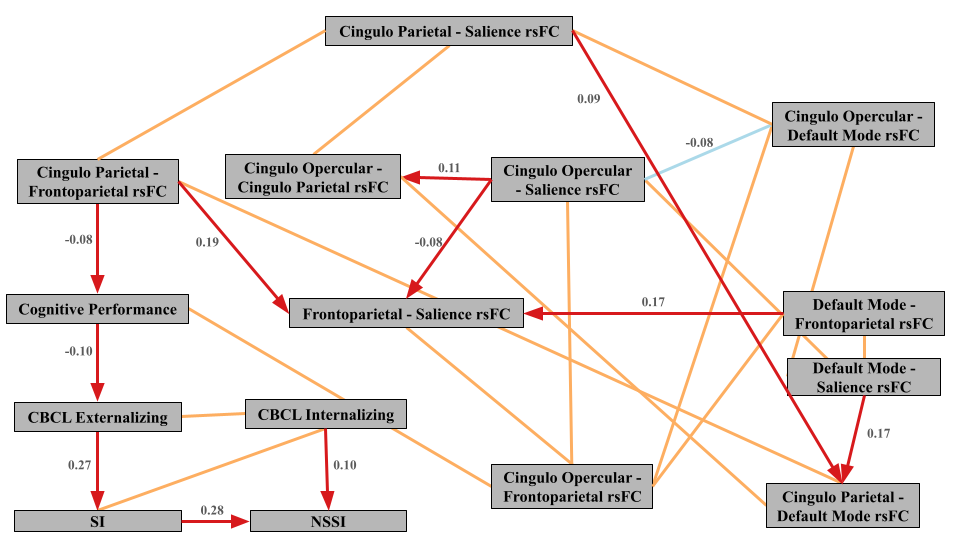
**

**Figure S2**. Red arrows indicate a directional causal relationship was identified, orange lines indicate that the two variables did not cause each other because at least one latent confounding variable was discovered, light blue lines indicate that either a causal relationship exists in one direction or a latent confounding variable was discovered, and dark blue lines indicate that the relationship between the two variables is unclear (i.e., there could be a directional causal relationship in either direction or there could be a latent confounding variable). Standardized edge weights calculated using structural equation modeling are displayed next to each edge. All edges were significant at the *p*=.01 level.

**Figure S3.** Full Causal Graph for the Female Sample Using Lifetime SITBs
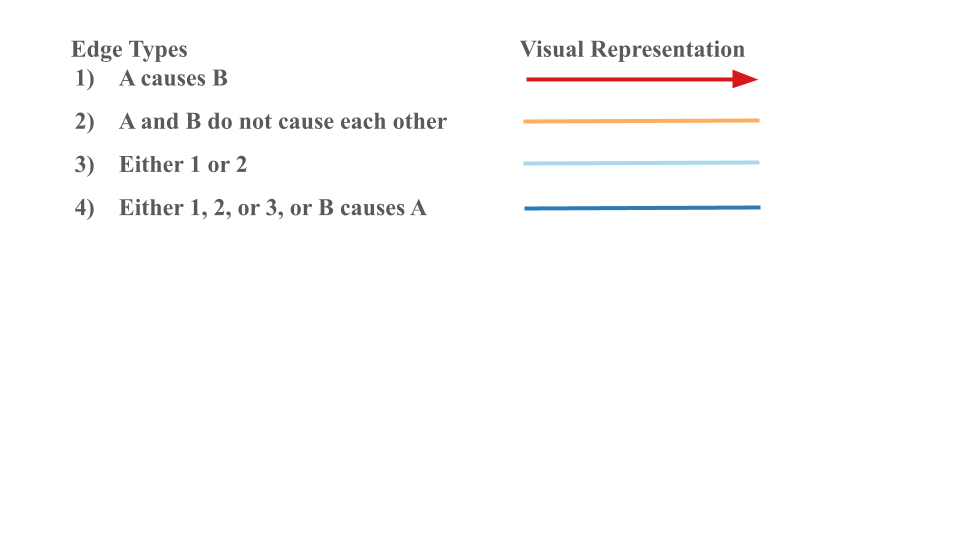


**
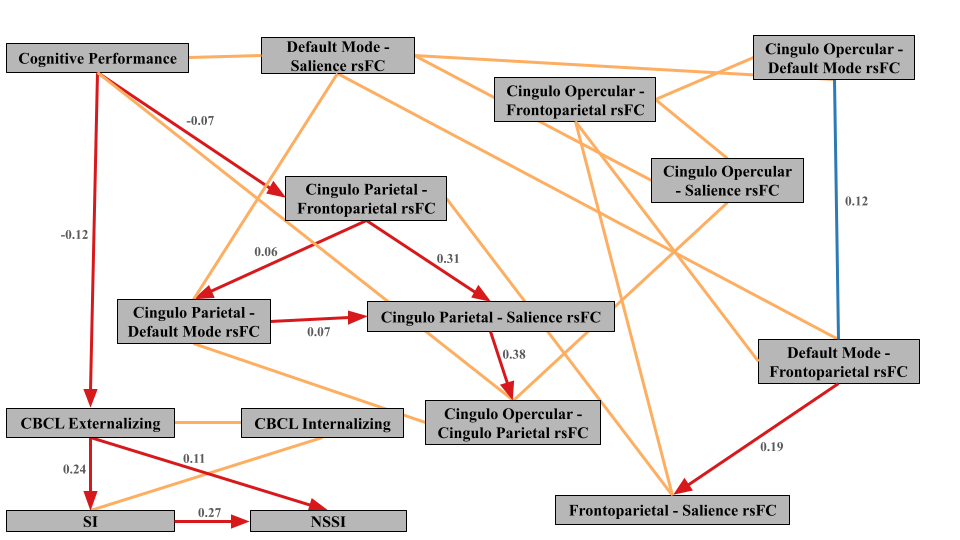
**

**Figure S3.** Red arrows indicate a directional causal relationship was identified, orange lines indicate that the two variables did not cause each other because at least one latent confounding variable was discovered, light blue lines indicate that either a causal relationship exists in one direction or a latent confounding variable was discovered, and dark blue lines indicate that the relationship between the two variables is unclear (i.e., there could be a directional causal relationship in either direction or there could be a latent confounding variable). Standardized edge weights calculated using structural equation modeling are displayed next to each edge. All edges were significant at the *p*=.01 level.
